# MR Image Reconstruction Based on Iterative Split Bregman Algorithm and Nonlocal Total Variation

**DOI:** 10.1155/2013/985819

**Published:** 2013-08-12

**Authors:** Varun P. Gopi, P. Palanisamy, Khan A. Wahid, Paul Babyn

**Affiliations:** ^1^Department of Electronics and Communication Engineering, National Institute of Technology, Tiruchirappalli, Tamil Nadu 620015, India; ^2^Department of Electrical and Computer Engineering, University of Saskatchewan, Saskatoon, SK, Canada; ^3^Department of Medical Imaging, Royal University Hospital, University of Saskatchewan, Saskatoon, SK, Canada

## Abstract

This paper introduces an efficient algorithm for magnetic resonance (MR) image reconstruction. The proposed method minimizes a linear combination of nonlocal total variation and least-square data-fitting term to reconstruct the MR images from undersampled *k*-space data. The nonlocal total variation is taken as the *L*_1_-regularization functional and solved using Split Bregman iteration. The proposed algorithm is compared with previous methods in terms of the reconstruction accuracy and computational complexity. The comparison results demonstrate the superiority of the proposed algorithm for compressed MR image reconstruction.

## 1. Introduction

Magnetic resonance (MR) imaging has been utilized in diagnosis because of its glorious depiction of soft tissue changes and noninvasive nature. As explained in [1, 2], it is possible to accurately reconstruct the MR images from undersampled *k*-space data using compressed sensing and considerably scale back the scanning period. Suppose *u* ∈ *R* ^*n*^ is a sparse signal, and let *K* be a measurement matrix such that *Ku* = *v*, where *v* is the observed data. Then, recovering *u* from *v* is equivalent to solve

(1) $$\begin{array}{c} \underset{u}{\min}J\left(u\right)\;\text{such}\;\;\text{that}\;\;Ku=v, \end{array}$$

where *J*(*u*) is a regularizing function; usually it may be bounded variation or Besov norm. In case of compressed sensing MRI, *K* is partial Fourier matrix (*K* = *Pℱ*), where *P* ∈ *R* ^*M*×*N*^ is an identity matrix (*M* ≪ *N*), *ℱ* is a discrete Fourier matrix, and *v* is the observed *k*-space data contaminated with Gaussian noise of variance *σ* ^2^, the relaxation form for (1) should be given by

(2) $$\begin{array}{c} \underset{u}{\min}J\left(u\right)\;\text{such}\;\;\text{that}\;\;{||Ku-v||}_{2}^{2}<\sigma^{2}. \end{array}$$

In order to make (1) simple to solve, first convert it into an unconstrained optimization problem. One common method for doing this is to use a penalty function/continuation method, which approximates (1) by a problem of the form

(3) $$\begin{array}{c} \underset{u}{\min}J\left(u\right)+\frac{\mu }{2}{||Ku-v||}_{2}^{2}, \end{array}$$

where *μ* is a balancing parameter between the fidelity term and sparsity term [3, 4]. This is the unconstrained problem we need to solve. 

There are a lot of iterative methods existing to reconstruct MR images from undersampled data such as conjugate gradient algorithm (CG) [4], operator splitting algorithm (TVCMRI) [5], variable splitting method (RecPF) [6], composite splitting algorithm (CSA) and its accelerated version called a fast composite splitting algorithm (FCSA) [7], and split Bregman algorithm combined with total variation (SB-TV) [8]. The Split Bregman method provides better solution to a wide class of *L*_1_-regularized problems. In SB-TV, the Split Bregman technique is applied to the Rudin-Osher-Fatemi functional to a compressed sensing problem that arises in a magnetic resonance imaging. A detailed explanation about the Split Bregman technique is given in Section 2. Reconstruction using CG is very slow for MR images. TVCMRI and RecPF can replace iterative linear solvers with Fourier domain computations, which can provide a reduction in computation time. FCSA performs much better than TVCMRI and RecPF and CSA. FCSA is based on wavelet sparsity and total variation (TV). However, despite the huge popularity of TV minimization, it has also some unwanted effects. In the presence of noise, it tends to piecewise constant solutions, the so called staircasing effect which was analyzed in detail in [9, 10]. Another deficit of TV regularization is the systematic loss of contrast in the reconstructions, even if the given data is noise free. This effect is the well-known systematic error of the total variation minimization and was studied extensively in [11, 12]. The major problem associated with TV-based compressed sensing method is that it tries to uniformly penalize the image gradient irrespective of the underlying image structures, and thus low contrast regions are sometimes over smoothed [13]. To resolve this issue, we propose a new algorithm which jointly minimizes a linear combination of nonlocal total variation and least-square data-fitting term to reconstruct the MR images from under-sampled data. In medical image reconstruction, fine structures, details, and texture should be preserved. The nonlocal total variation makes the recovered image quality sharper, and they preserve the fine structures, details, and textures. NLTV is much better than TV for improving the signal-to-noise ratio in practical application [14–16]. Authors of [17, 18] showed that among the existing nonlocal regularization techniques, NLTV is performing well in reconstruction. So in the proposed method, the nonlocal total variation is taken as the *L*_1_-regularization functional and solved using Split Bregman algorithm. Numerous experiments have been done on real MR images to show the efficiency and advantages of the proposed work in terms of computational complexity and reconstruction accuracy.

## 2. Split Bregman Algorithm and Regularization Method

The *L*_1_-regularized problems have many important applications in engineering and imaging science. The general form of such problems is

(4) $$\begin{array}{c} \underset{u}{\min}\left|\Phi \left(u\right)\right|+L\left(u\right), \end{array}$$

where |·| represents the *L*_1_-norm and both Φ(*u*) and *L*(*u*) are convex functions. Many important problems in image processing can be interpreted as *L*_1_-regularized optimization problems. Equation (3) is an example of such a problem

(5) $$\begin{array}{c} \underset{u}{\min}J\left(u\right)+\frac{\mu }{2}{||Ku-v||}_{2}^{2}. \end{array}$$

Unfortunately, for several issues, selecting a large value for *μ* makes (5) extremely tough to solve numerically [19, 20]. Also, for several applications, *μ* should be increased in small steps, creating the strategy less efficient. Bregman iteration can be used to reduce (5) into small sequence of unconstrained problems for further processing.

### 2.1. Bregman Iteration

Bregman iteration is a method for finding extrema of convex functionals [21]. Bregman iteration was already applied to solve the basis pursuit problem in [22] and medical imaging problem in [23]. The general formulation of this method is explained by using Bregman distance. The Bregman distance associated with a convex function *J* at the point *v* is

(6) $$\begin{array}{c} D_{J}^{p}\left(u,v\right)=J\left(u\right)-J\left(v\right)-\langle p,u-v\rangle , \end{array}$$

where *p* is in the subgradient of *J* at *v*. Clearly, this is not a distance in the usual sense, it measures closeness in the sense that *D*_*J*_^*p*^(*u*, *v*) ≥ 0 and *D*_*J*_^*p*^(*u*, *v*)≥*D*_*J*_^*p*^(*w*, *v*) for *w* on the line segment between *u* and *v*. Again, consider two convex energy functionals, *J* and *L*, defined over *R* ^*n*^ with min_*u*∈*R*^*n*^_ *L*(*u*) = 0. The associated unconstrained minimization problem is

(7) $$\begin{array}{c} \underset{u}{\min}J\left(u\right)+\mu L\left(u\right). \end{array}$$

Modify this problem by iteratively solving

(8) $$\begin{array}{c} u^{k+1}=\underset{u}{\min}D_{J}^{p}\left(u,u^{k}\right)+\mu L\left(u\right), \end{array}$$

(9) $$\begin{array}{c} u^{k+1}=\underset{u}{\min}J\left(u\right)-\langle p^{k},u-u^{k}\rangle +\mu L\left(u\right) \end{array}$$

as suggested by Bregman in [21]. For simplicity, assume that *L* is differentiable, and in this case, we have 0 ∈ *δ*(*D*_*J*_^*p*^(*u*, *u* ^*k*^) + *μL*(*u*)), where this subdifferential is evaluated at *u* ^*k*+1^. Since *p* ^*k*+1^ ∈ *δJ*(*u* ^*k*+1^) at this location, then

(10) $$\begin{array}{c} p^{k+1}=p^{k}-\nabla L\left(u^{k+1}\right). \end{array}$$

Apply the Bregman iteration (8) on (5)

(11) $$\begin{array}{c} u^{k+1}=\underset{u}{\min}D_{J}^{p}\left(u,u^{k}\right)+\frac{\mu }{2}{||Ku-v||}_{2}^{2},\\ u^{k+1}=\underset{u}{\min}J\left(u\right)-\langle p^{k},u-u^{k}\rangle +\frac{\mu }{2}{||Ku-v||}_{2}^{2},\\ p^{k+1}=p^{k}-\mu K^{T}\left(Ku^{k+1}-v\right). \end{array}$$

Bregman iterations of this form were considered in [12]. Here, it is shown that when *K* is linear, this seemingly complicated iteration is equivalent to the simplified method

(12) $$\begin{array}{c} u^{k+1}=\underset{u}{\min}J\left(u\right)+\frac{\mu }{2}{||Ku-v^{k}||}_{2}^{2},\\ v^{k+1}=v^{k}+v-Ku^{k+1}. \end{array}$$

### 2.2. Split Bregman Method

Apply Bregman framework to solve *L*_1_-regularized problem (4). In this case, assume that both Φ(*u*) and *L*(*u*) are convex functions and Φ(*u*) is differentiable. In this method, decouple the *L*_1_ and *L*_2_ parts of energy in (4). Now, consider (4) as

(13) $$\begin{array}{c} \underset{u,d}{\min}\left|d\right|+L\left(u\right)\;\text{such}\;\;\text{that}\;\;d=\Phi \left(u\right). \end{array}$$

To solve this problem, first convert it into an unconstrained problem

(14) $$\begin{array}{c} \underset{u,d}{\min}\left|d\right|+L\left(u\right)+\frac{\mu }{2}{||d-\Phi \left(u\right)||}_{2}^{2}. \end{array}$$

Let *J*(*u*, *d*) = |*d* | +*L*(*u*), and let *K*(*u*, *d*) = *d* − Φ(*u*), then we can see that (14) is simply an application of (5). Use (11) to obtain the solution for the above problem:

(15) (uk+1,dk+1)=min⁡u,dDJp(u,uk,d,dk)+μ2||d−Φ(u)||22,(uk+1,dk+1)=min⁡u,dJ(u,d)−〈puk,u−uk〉−〈pdk,d−dk〉   +μ2||d−Φ(u)||22,puk+1=puk−μ(∇Φ)T(Φuk+1−dk+1),pdk+1=pdk−μ(dk+1−Φuk+1).

Applying simplification presented in (12), we get the following solutions:

(16) $$\begin{array}{c} \left(u^{k+1},d^{k+1}\right)=\underset{u,d}{\min}\left|d\right|+L\left(u\right)+\frac{\mu }{2}{||d-\Phi \left(u\right)-v^{k}||}_{2}^{2}, \end{array}$$

(17) $$\begin{array}{c} v^{k+1}=v^{k}+\left(\Phi \left(u^{k+1}\right)-d^{k+1}\right). \end{array}$$

In order to perform the minimization effectively, split the *L*_1_ and *L*_2_ components of (16) and minimize with respect to *u* and *d* separately. The two steps result in the following solutions:

(18) $$\begin{array}{c} u^{k+1}=\underset{u}{\min}L\left(u\right)+\frac{\mu }{2}{||d^{k}-\Phi \left(u\right)-v^{k}||}_{2}^{2}, \end{array}$$

(19) $$\begin{array}{c} d^{k+1}=\underset{d}{\min}\left|d\right|+\frac{\mu }{2}{||d-\Phi \left(u^{k+1}\right)-v^{k}||}_{2}^{2}. \end{array}$$

In (19), there is no coupling between elements of *d*. We can use shrinkage operator to find the optimal value of *d* as follows:

(20) $$\begin{array}{c} d^{k+1}=\text{shrink}\left(\Phi {\left(u\right)}_{j}+v_{j}^{k},\frac{1}{\mu }\right), \end{array}$$

where

(21) $$\begin{array}{c} \text{shrink}\left(x,\lambda \right)=\frac{x}{\left|x\right|}*\max\left(\left|x\right|-\lambda ,0\right). \end{array}$$

This shrinkage operation is extremely fast and requires only few operations per element of *d* ^*k*+1^.

Based on the above described equations, the generalized Split Bregman algorithm can be explained in Algorithm 1.

### 2.3. Nonlocal Total Variation Norm

Total Variation (TV) regularization [24, 25] makes the recovered image quality sharper, but they do not preserve the fine structures, details, and textures. This effect is caused by the regularity assumption of the TV formulation of the image model, namely, that the image has a simple geometric description consisting of a set of connected sets (objects) with smooth contours (edges). Additionally, the model assumes that the image is smooth inside single objects and has discontinuous jumps across the boundaries. Therefore, TV regularization is optimal to reduce the noise and to reconstruct the main geometrical configuration in an image. However, it fails to preserve texture, details, and fine structures, because they behave in all aspects like noise and thus cannot be distinguished from noise. The nonlocal total variation (NLTV) extends the TV functional to a nonlocal variant using the definition of nonlocal derivative operators based on a nonlocal weight function [14–17, 26, 27]. NLTV is an effective tool instead of TV for improving the signal-to-noise ratio in practical application [14–16]. Recently, it has been successfully used for 4D computed tomography reconstruction from few projection data [28]. NLTV extends the TV functional to a nonlocal variant using the definition of nonlocal derivative operators based on a nonlocal weight function (graph). The notion nonlocal means that any point can directly interact with any other point in the whole image domain, where the intensity of the interaction is depending on the value of the weight function. This weight function should represent the similarity of the two points and should be significant, if both points are similar in an appropriate measure. Therefore, the expectation is that such an approach is able to process both structures (geometrical parts) and texture within the same framework, due to the identification of recurring structures in the whole image. A brief review regarding the definition of nonlocal functional is given below.

Let *Ω* ∈ *R* ^2^, and let *u* :*Ω* → *R* be a real function. Assume that *w* : *Ω* × *Ω* → *R* is a weight function which is symmetric and nonnegative. Then, the nonlocal gradient of an image *u*(*x*) is defined as

(22) $$\begin{array}{c} \nabla_{\text{NL}}u\left(x,y\right):=\left(u\left(x\right)-u\left(y\right)\right)\sqrt{w\left(x,y\right)}:\Omega \times \Omega \to R. \end{array}$$

The norm of a vector *q* : *Ω* × *Ω* → *R* at point *x* ∈ *Ω* is given by

(23) $$\begin{array}{c} \left|q\right|\left(x\right)=\sqrt{\int_{\Omega }q{\left(x,y\right)}^{2}dy.} \end{array}$$

Hence, the norm of the nonlocal gradient of a function *u* : *Ω* → *R* at point *x* ∈ *Ω* is represented as

(24) $$\begin{array}{c} \left|\nabla_{\text{NL}}u\right|\left(x\right)=\sqrt{\int_{\Omega }{\left(u\left(x\right)-u\left(y\right)\right)}^{2}w\left(x,y\right)dy}:\Omega \to R_{+}. \end{array}$$

The nonlocal divergence operator can be defined using the standard adjoint relation with the nonlocal gradient as follows:

(25) 〈∇NLu,q〉:=−〈u,divNLq〉, ∀u:Ω→R,q:Ω×Ω→R

which guides to the definition of nonlocal divergence of the nonlocal vector *q*

(26) $$\begin{array}{c} \text{di}{\text{v}}_{\text{NL}}q\left(x\right)=\int_{\Omega }\left(q\left(x,y\right)-q\left(y,x\right)\right)\sqrt{w\left(x,y\right)}dy:\Omega \to R. \end{array}$$

Next, the nonlocal Laplacian operator is defined as

(27) ΔNLu(x):=12divNL(∇NLu(x))=∫Ω(u(x)−u(y))w(x,y)dy.

The discrete forms of the nonlocal gradient, divergence, and laplace operators can be represented as follows:

(28) $$\begin{array}{c} {\left|\nabla_{\text{NL}}u\right|}_{i}:=\sqrt{\underset{j}{\sum }{\left(u_{j}-u_{i}\right)}^{2}w_{ij}},\\ {\left(\text{di}{\text{v}}_{\text{NL}}q\right)}_{i}:=\underset{j}{\sum }\left(q_{i,j}-q_{j,i}\right)\sqrt{w_{ij}},\\ {\left(\Delta_{\text{NL}}u\right)}_{i}:=\underset{j}{\sum }\left(u_{j}-u_{i}\right)w_{ij}, \end{array}$$

where *u*_*i*_ represents the value of a pixel *i* in the image (1 ≤ *i* ≤ *N*) and *w*_*ij*_ is the discrete sparse version of *w*(*x*, *y*), and it is defined as

(29) $$\begin{array}{c} w\left(x,y\right)=\exp\left\{-\frac{G_{\sigma }*\left({||u\left(x+\cdot \right)-u\left(y+\cdot \right)||}^{2}\right)}{2h^{2}}\right\}, \end{array}$$

where *h* is a filtering parameter; in general *h* corresponds to the noise level; usually, we set it to be the standard deviation of the noise, and *G*_*σ*_ is a Gaussian of standard deviation *σ*, and *u*(*x*) and *u*(*y*) are the image values in pixel *x* and *y*. The weight functions *w*(*x*, *y*) denote how much the difference between pixels *x* and *y* is penalized in the images. The more similar the neighborhoods of *x* and *y* are, more the difference should be penalized, and vice versa. The weight functions *w*(*x*, *y*), significant only if the window around *y* looks like the corresponding window around *x*. Hence, the nonlocal algorithm is very efficient in reducing noise, while preserving contrast in natural images and redundant structures such as texture. In our work, we used 5 × 5 pixel patches, a search neighborhood window of size 11 × 11. The observed noisy data *v* is taken as the reference image to construct the weight, and by this weighed averaging, the structures, for example, boundaries, are reinforced, while the noise gets cancelled out [29]. The weights are computed by using either a distance between the noisy pixel values |*u*(*x*) − *u*(*y*)| [30–32] or a distance between the patches around *x* and *y* [33–35]. The use of NLTV reduces the noise in the reconstructed image, thus the difference between reference and reconstructed image reduces. From the definition of signal-to-noise ratio (SNR), it is clear that the reduction in image difference increases the SNR. 

## 3. Proposed Work

The proposed work jointly minimizes a linear combination of nonlocal total variation and least-square data-fitting term to reconstruct the MR image from undersampled data. The main aim is to solve the compressed sensing MRI problem (2) using Split Bregman algorithm and nonlocal total variation. In this work, the nonlocal total variation is taken as the *L*_1_-regularization functional and solved using Split Bregman iteration. Recall (2)

(30) $$\begin{array}{c} \underset{u}{\min}J\left(u\right)\;\text{such}\;\;\text{that}\;\;{||Ku-v||}_{2}^{2}<\sigma^{2}. \end{array}$$

Using Bregman iteration method, (30) can be reduced to a sequence of unconstrained problems of the form

(31) $$\begin{array}{c} u^{k+1}=\underset{u}{\min}J\left(u\right)+\frac{\mu }{2}{||Ku-v^{k}||}_{2}^{2}, \end{array}$$

(32) $$\begin{array}{c} v^{k+1}=v^{k}+v-Ku^{k+1}, \end{array}$$

where *J*(*u*) represents *L*_1_-regularization term. In order to proceed further selection of regularization method is important. Here, we choose nonlocal total variation (NLTV) as the regularizer; that is

(33) $$\begin{array}{c} J\left(u\right)=\left|\nabla_{\text{NL}}u\right|. \end{array}$$

Now, (31) becomes

(34) $$\begin{array}{c} \underset{u}{\min}\;{||\nabla_{\text{NL}}u||}_{1}+\frac{\mu }{2}{||Ku-v^{k}||}_{2}^{2}, \end{array}$$

where ||∇_NL_ *u*||_1_ = ∑_*i*_ | ∇_NL_ *u*|_*i*_. We can write (34) as follows by introducing an auxiliary variable *d* instead of ∇_NL_ *u*:

(35) $$\begin{array}{c} \underset{u,d}{\min}\;{||d||}_{1}+\frac{\mu }{2}{||Ku-v^{k}||}_{2}^{2}\;\text{such}\;\;\text{that}\;\;d=\nabla_{\text{NL}}u. \end{array}$$

Equation (35) can be converted into unconstrained form by using the quadratic penalty method

(36) $$\begin{array}{c} \underset{u,d}{\min}{||d||}_{1}+\frac{\gamma }{2}{||d-\nabla_{\text{NL}}u||}_{2}^{2}+\frac{\mu }{2}{||Ku-v^{k}||}_{2}^{2}. \end{array}$$

Using split Bregman method, (36) can be transformed into the following forms:

(37) $$\begin{array}{c} \underset{u,d}{\min}{||d||}_{1}+\frac{\gamma }{2}{||d-\nabla_{\text{NL}}u-b^{k}||}_{2}^{2}+\frac{\mu }{2}{||Ku-v^{k}||}_{2}^{2}, \end{array}$$

(38) $$\begin{array}{c} b^{k+1}=b^{k}+\nabla_{\text{NL}}u^{k+1}-d^{k+1}. \end{array}$$

Equation (37) is convex and can be minimized by alternatively solving the following two minimization subproblems with respect to *u* and *d*

(39) $$\begin{array}{c} u^{k+1}=\;\underset{u}{\arg\min}\;\frac{\gamma }{2}{||d^{k}-\nabla_{\text{NL}}u-b^{k}||}_{2}^{2}+\frac{\mu }{2}{||Ku-v^{k}||}_{2}^{2},\\ d^{k+1}=\;\underset{d}{\arg\min}{\;||d||}_{1}+\frac{\gamma }{2}{||d-\nabla_{\text{NL}}u-b^{k}||}_{2}^{2}. \end{array}$$

By direct computation, the optimal conditions of (39) are

(40) $$\begin{array}{c} -\gamma \text{di}{\text{v}}_{\text{NL}}\left(d^{k}-\nabla_{\text{NL}}u-b^{k}\right)+\mu K^{T}\left(Ku-v^{k}\right)=0, \end{array}$$

(41) $$\begin{array}{c} d^{k+1}=\frac{\nabla_{\text{NL}}u^{k+1}+b^{k}}{\left|\nabla_{\text{NL}}u^{k+1}+b^{k}\right|}\max\left(\left|\nabla_{\text{NL}}u^{k+1}+b^{k}\right|-\frac{1}{\gamma },0\right). \end{array}$$

Use the Gauss-Seidel iteration to get a fast solution of (40), and the discrete solution is represented as

(42) uik+1=1KTKμ+γ∑jwij ×(γ∑jwijuk+μvik+ γ∑jwij(dijk−bijk−djik+bjik)).

As explained in the introduction, *K* is partial Fourier matrix (*K* = *Pℱ*), where *P* ∈ *R* ^*M*×*N*^ is an identity matrix (*M* ≪ *N*) and *ℱ* is a discrete Fourier matrix. Using the identity *ℱ* ^*T*^ = *ℱ* ^−1^, now (42) becomes

(43) uik+1=1ℱ−1PTPℱμ+γ∑jwij ×(γ∑jwijuk+μvik+γ∑jwij(dijk−bijk−djik+bjik)).

The discrete solution for (41) can be written as

(44) di,jk+1=wij(ujk+1−uik+1)+bijk∑jwij(ujk+1−uik+1)2+(bijk)2 ×max⁡(∑jwij(ujk+1−uik+1)2+(bijk)2−1γ,0).

Finally, the Bregman variable is updated as

(45) $$\begin{array}{c} b_{ij}^{k+1}=b_{ij}^{k}+\underset{j}{\sum }\sqrt{w_{ij}}\left(u_{j}^{k+1}-u_{i}^{k+1}\right)-d_{ij}^{k+1}. \end{array}$$

The proposed method is summarized as Algorithm 2.

## 4. Evaluation of Image Quality

In this work, a detailed evaluation study has done on the reconstruction of MR images, which represent varying degrees of object structural complexity. Even though algorithms based on regularization techniques effectively remove streaks, other aspects of image quality should also be analyzed. To address this, a number of image quality evaluations are performed at different levels including qualitative visualization-based evaluation and quantitative metric-based evaluation. 

### 4.1. Qualitative Visualization-Based Evaluation

In qualitative visualization-based evaluation, reconstructed image obtained with different algorithms are visually compared with the reference image. 

### 4.2. Quantitative Metric-Based Evaluation

Besides the visualization-based evaluation, similarity between reconstructed and reference images is quantitatively assessed by means of four measures such as signal-to-noise ratio (SNR), relative error (RE), structural similarity index (SSIM), and feature similarity index (FSIM). SNR and RE are widely used for measuring reconstruction accuracy, SSIM and FSIM are used for evaluating the similarity between reconstructed and reference image.

#### 4.2.1. Signal-to-Noise Ratio (SNR)

One can see that

(46) $$\begin{array}{c} \text{SNR}=10\log\frac{\;||u_{\text{ref}}-\hat{u}||}{||u_{\text{ref}}-u_{\text{rec}}||}, \end{array}$$

where *u*_ref_ is the reference image, $\hat{u}$ is the mean intensity value of *u*_ref_, and *u*_rec_ is the reconstructed image. 

#### 4.2.2. Relative Error (RE)

One can see that

(47) $$\begin{array}{c} \text{RE}=\frac{||u_{\text{rec}}-u_{\text{ref}}||}{||u_{\text{ref}}||}\times 100\%. \end{array}$$

#### 4.2.3. Structural Similarity Index (SSIM)

The SNR measurement gives a numerical value on the damage, but it does not describe its type. Moreover, as is noted in [36, 37], it does not quite represent the quality perceived by human observers. For medical imaging applications, where images are degraded, must eventually be examined by experts, traditional evaluation remains insufficient. For this reason, objective approaches are needed to assess the medical imaging quality. We then evaluate a new paradigm to estimate the quality of medical images based on the assumption that the human visual system (HVS) is highly adapted to extract structural information. The similarity index compares the brightness *I*(*x*, *y*), contrast *c*(*x*, *y*), and structure *s*(*x*, *y*) between each pair of vectors, where the SSIM index between two signals *x* and *y* is given by the following expression [38, 39]:

(48) $$\begin{array}{c} \text{SSIM}\left(x,y\right)=I\left(x,y\right)c\left(x,y\right)s\left(x,y\right). \end{array}$$

However, the comparison of brightness is determined by the following expression:

(49) $$\begin{array}{c} I\left(x,y\right)=\frac{2\mu_{x}\mu_{y}+C_{1}}{\mu_{x}+\mu_{y}+C_{1}}, \end{array}$$

where the average intensity of signal *x* is given by

(50) $$\begin{array}{c} \mu_{x}=\frac{1}{N}\overset{N}{\underset{i=1}{\sum }}x_{i},\;\;C_{1}={\left(K_{1}L\right)}^{2} \end{array}$$

the constant *K*_1_ ≪ 1, and *L* is the dynamic row of the pixel values (255 for an image coded on 8 bits). The function of contrast comparison takes the following form:

(51) $$\begin{array}{c} c\left(x,y\right)=\frac{2\sigma_{x}\sigma_{y}}{\sigma_{x}^{2}+\sigma_{y}^{2}+C_{2}}, \end{array}$$

where $\sigma_{x}=\sqrt{\mu_{x}(x^{2})-\mu_{x}^{2}(x)}$ is the standard deviation of the original signal *x*, *C*_2_ = (*K*_2_ *L*)^2^, and the constant *K*_2_ ≪ 1.

The function of structure comparison is defined as follows:

(52) $$\begin{array}{c} s\left(x,y\right)=\frac{\sigma_{xy}+C_{3}}{\sigma_{x}\sigma_{y}+C_{3}}=\frac{\mathrm{cov}\left(x,y\right)+C_{3}}{\sigma_{x}\sigma_{y}+C_{3}}, \end{array}$$

where cov⁡(*x*, *y*) = *μ*_*xy*_ − *μ*_*x*_ *μ*_*y*_ and *C*_3_ = *C*_2_/2.

Then, the expression of the structural similarity index becomes

(53) $$\begin{array}{c} \text{SSIM}\left(x,y\right)=\frac{\left(2\mu_{x}\mu_{y}+C_{1}\right)\left(2\sigma_{xy}+C_{2}\right)}{\left(\mu_{x}^{2}+\mu_{y}^{2}+C_{1}\right)\left(\sigma_{x}^{2}+\sigma_{y}^{2}+C_{2}\right)}. \end{array}$$

#### 4.2.4. Feature Similarity Index (FSIM)

SSIM index provides image quality assessment (IQA) from pixel-based stage to structure-based stage. Human visual system (HVS) understands an image mainly based on its low-level features: mainly, the phase congruency (PC), which is a measure of the significance of a local structure and it is dimensionless. PC is used as the primary feature in FSIM [40]. The secondary feature used in FSIM is the image gradient magnitude (GM). In order to find out the feature similarity between two images *f*_1_ and *f*_2_, the above mentioned parameters PC and GM are to be calaculated first.

Let PC_1_, PC_2_, *G*_1_, and *G*_2_ be the phase congruency and gradient magnitude of images *f*_1_ and *f*_2_, respectively. Initially, separate the feature similarity measurement between *f*_1_(*x*) and *f*_2_(*x*) into two components. First similarity measure is based on PC_1_(*x*) and PC_2_(*x*) and is defined as

(54) $$\begin{array}{c} S_{\text{PC}}\left(x\right)=\frac{2\text{P}{\text{C}}_{1}\left(x\right)\cdot \text{P}{\text{C}}_{2}\left(x\right)+T_{1}}{\text{P}{\text{C}}_{1}^{2}\left(x\right)+\text{P}{\text{C}}_{2}^{2}\left(x\right)+T_{1}}, \end{array}$$

where *T*_1_ is a positive constant which increases the stability of *S*_PC_. Value of *T*_1_ depends on the dynamic range of PC. Similarly, the similarity measure based on GM values *G*_1_(*x*) and *G*_2_(*x*) is defined as

(55) $$\begin{array}{c} S_{G}\left(x\right)=\frac{2G_{1}\left(x\right)\cdot G_{2}\left(x\right)+T_{2}}{G_{1}^{2}\left(x\right)+G_{2}^{2}\left(x\right)+T_{2}}, \end{array}$$

where *T*_1_ is a positive constant which depends on the dynamic range of GM value.

Next step is to combine *S*_PC_(*x*) and *S*_*G*_(*x*) to get the similarity measure *S*_*L*_(*x*) between *f*_1_(*x*) and *f*_2_(*x*) and is defined as

(56) $$\begin{array}{c} S_{L}\left(x\right)={\left[S_{\text{PC}}\left(x\right)\right]}^{\alpha }\cdot {\left[S_{G}\left(x\right)\right]}^{\beta }. \end{array}$$

The relative importance of PC and GM features can be adjusted by means of the parameters *α* and *β*. For simplicity, set *α* = *β* = 1, then (56) becomes

(57) $$\begin{array}{c} S_{L}\left(x\right)=S_{\text{PC}}\left(x\right)\cdot S_{G}\left(x\right), \end{array}$$

where *S*_*L*_(*x*) represents the similarity at each location *x*, and the overall similarity should be found. For a given location *x*, if any of *f*_1_(*x*) and *f*_2_(*x*) has a significant PC value, it implies that this position *x* will have a high impact on HVS in evaluating the similarity between *f*_1_ and *f*_2_. Therefore, introducing a new term PC_*m*_(*x*) = max⁡(PC_1_(*x*), PC_2_(*x*)) to weight the importance of *S*_*L*_(*x*) in the overall similarity between *f*_1_ and *f*_2_. Accordingly, the FSIM index can defined as

(58) $$\begin{array}{c} \text{FSIM}=\frac{\sum_{x\in \Omega }S_{L}\left(x\right)\cdot \text{P}{\text{C}}_{m}\left(x\right)}{\sum_{x\in \Omega }\text{P}{\text{C}}_{m}\left(x\right)}, \end{array}$$

where *Ω* means the whole image spatial domain.

## 5. Experiments and Numerical Results

The experimental setup used in previous works [5–7] is explained here. Suppose that an MR image *u* has *m* × *m* pixels and the partial Fourier transform *K* in (3) consists of *n* rows of a *m* × *m* matrix corresponding to the full 2D discrete Fourier transform. The *n* selected rows correspond to the acquired data *v*. The sampling ratio is outlined as *n*/*m*. The scanning time is shorter if the sampling ratio is smaller. In the *k*-space, randomly choose more samples in low frequencies and fewer samples in higher frequencies. This sample theme has been widely used for compressed MR image reconstruction, and therefore similar themes are utilized in [4–7]. Practically, the sampling scheme and speed in MR imaging also depend on the physical and physiological limitations [4]. In the proposed work, the compressive sensing matrix *K* = *Pℱ*, where *P* is a row selector matrix, and *ℱ* is the Fourier transform matrix. For an *N* × *N* image, we randomly choose m coefficients, then *R* is a sampling matrix of size *m* × *N* ^2^. All experiments are conducted on a PC with an Intel core-i72670, 2.2 GHz CPU in MATLAB environment. The result of the proposed work is compared with the existing methods like TVCMRI [5], RecPF [6], CSA, FCSA [7], and SB-TV [8]. The observation measurement *v* is synthesized as *v* = *Ku* + *n*, where *n* is the noise, *K* and *v* are given as inputs, and *u* is the unknown target. In this work we considered two sets of observations: one is contaminated with Gaussian noise of standard deviation *σ* = 0.01, and the other is contaminated with Rician noise of noise level 5%.

**Figure 1:**
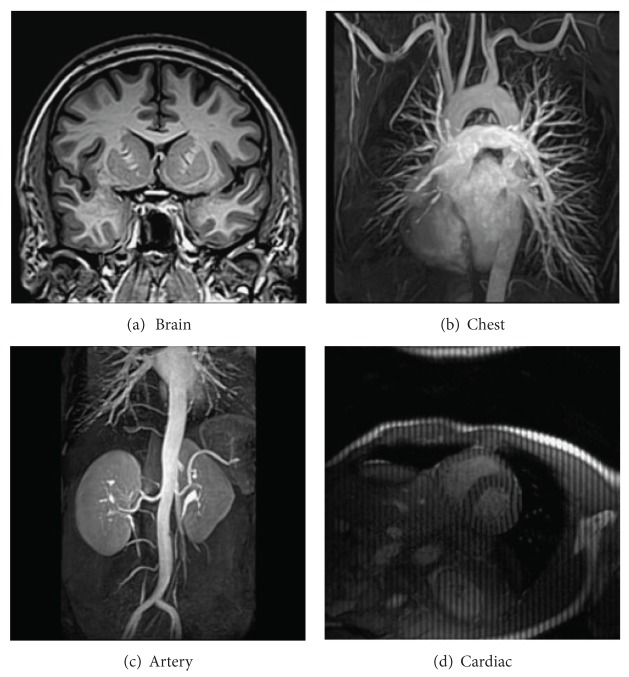
2D MR test images.

**Figure 2:**
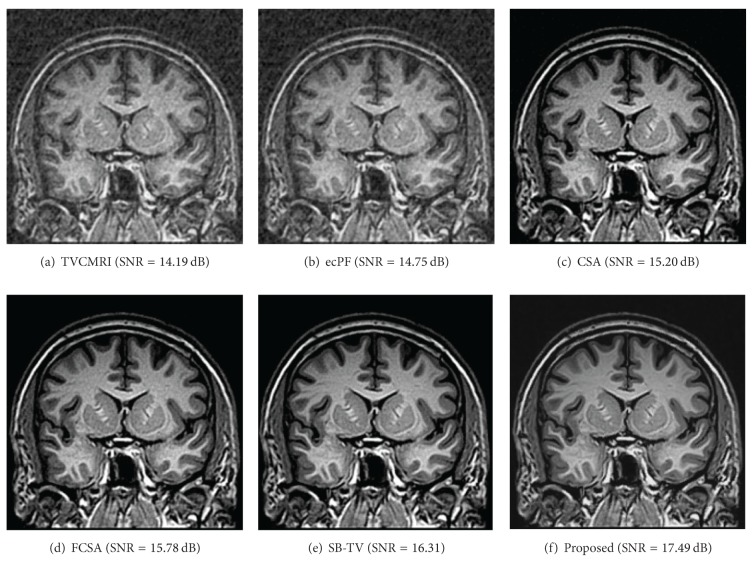
Brain MR image reconstruction from 20% samples (Gaussian noise case).

**Figure 3:**
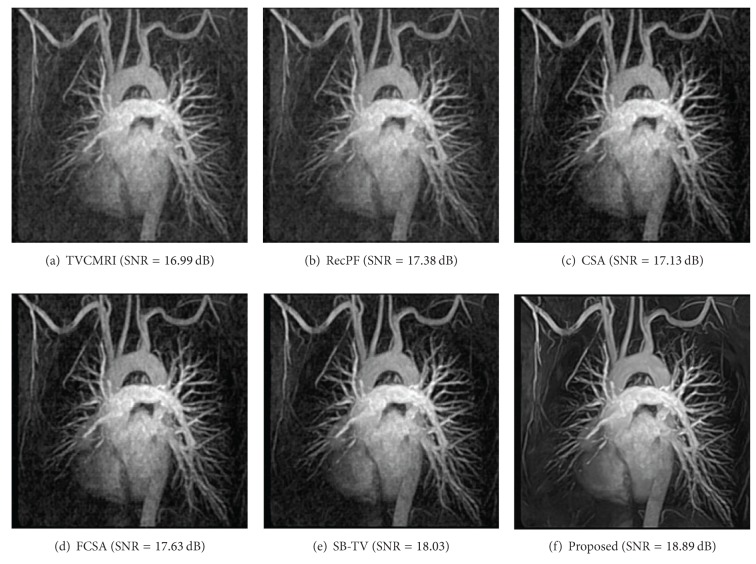
Chest MR image reconstruction from 20% samples (Gaussian noise case).

**Figure 4:**
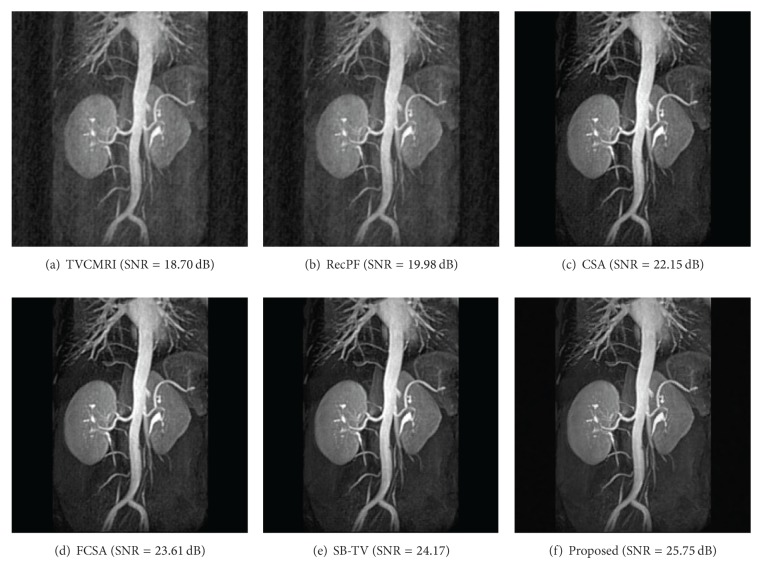
Artery MR image reconstruction from 20% samples (Gaussian noise case).

**Figure 5:**
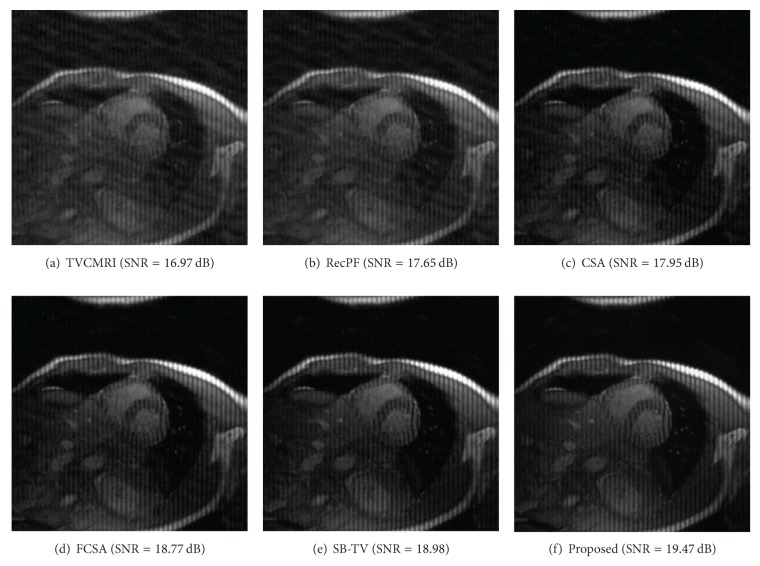
Cardiac MR image reconstruction from 20% samples (Gaussian noise case).

**Figure 6:**
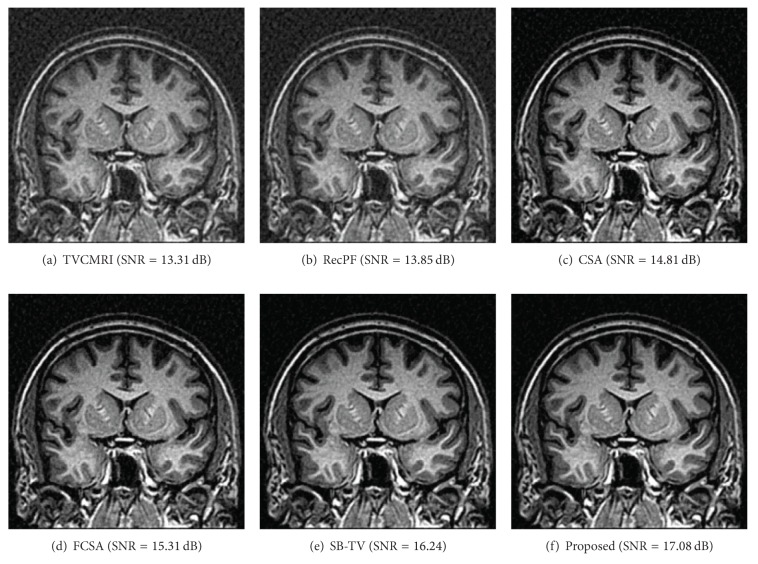
Brain MR image reconstruction from 20% samples (Rician noise case).

**Figure 7:**
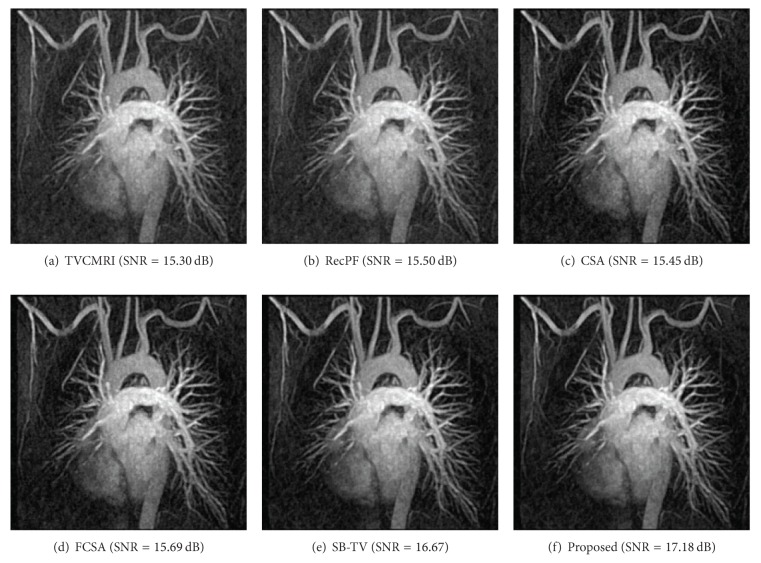
Chest MR image reconstruction from 20% samples (Rician noise case).

**Figure 8:**
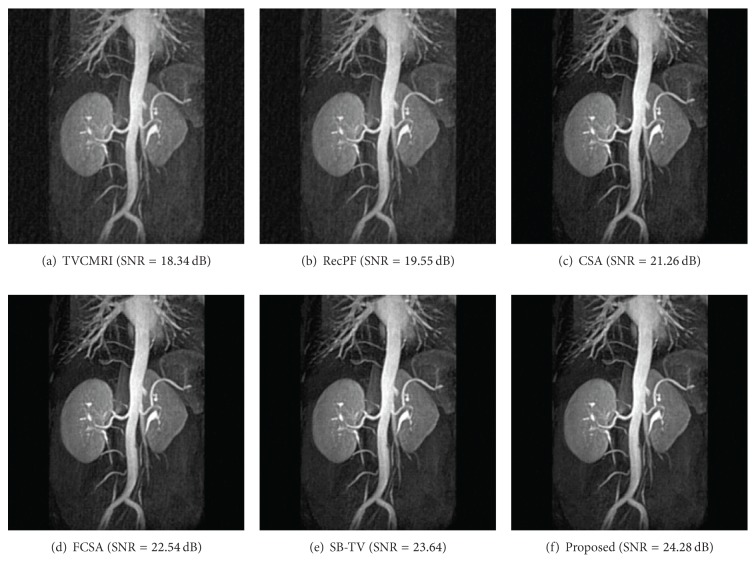
Artery MR image reconstruction from 20% samples (Rician noise case).

**Figure 9:**
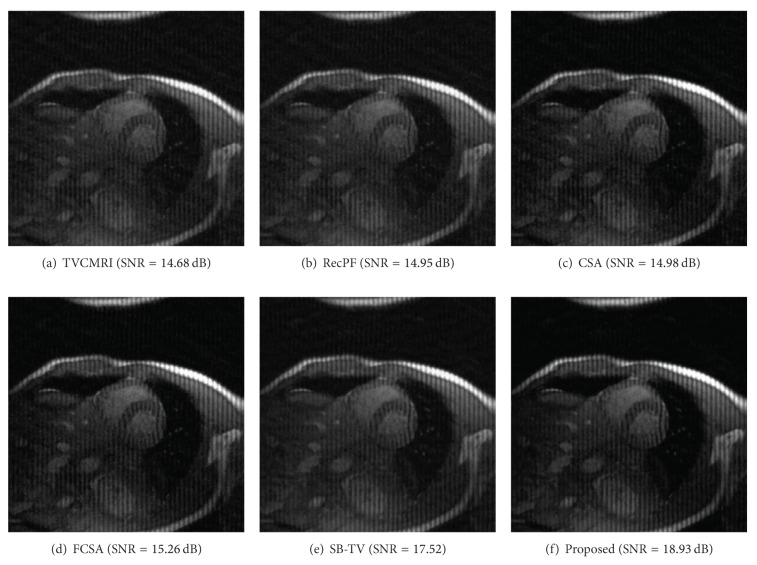
Cardiac MR image reconstruction from 20% samples (Rician noise case).

**Figure 10:**
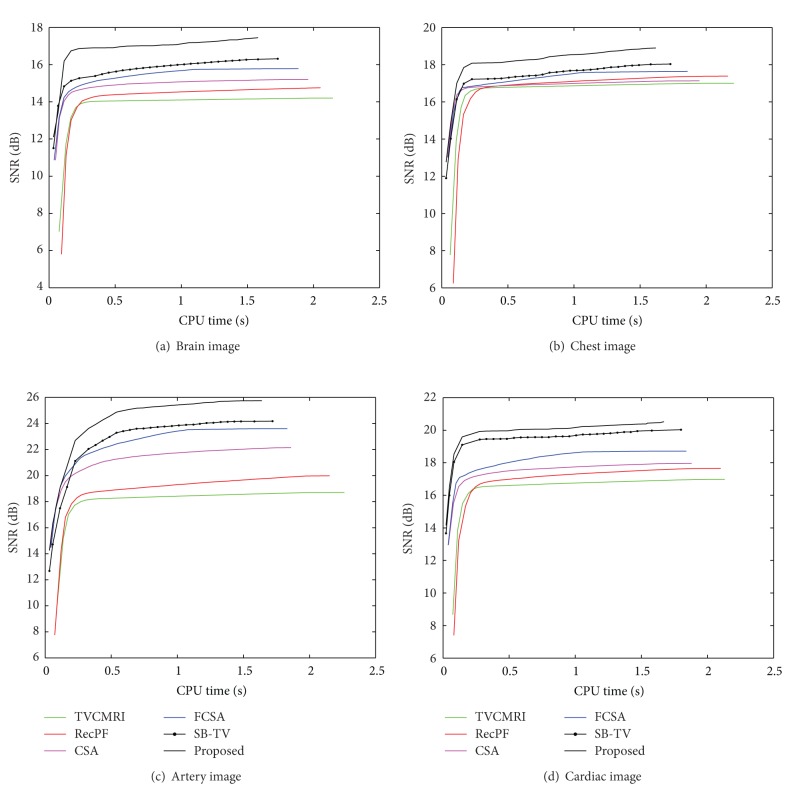
Performance comparisons (CPU time versus SNR) on different MR images (Gaussian noise case).

**Figure 11:**
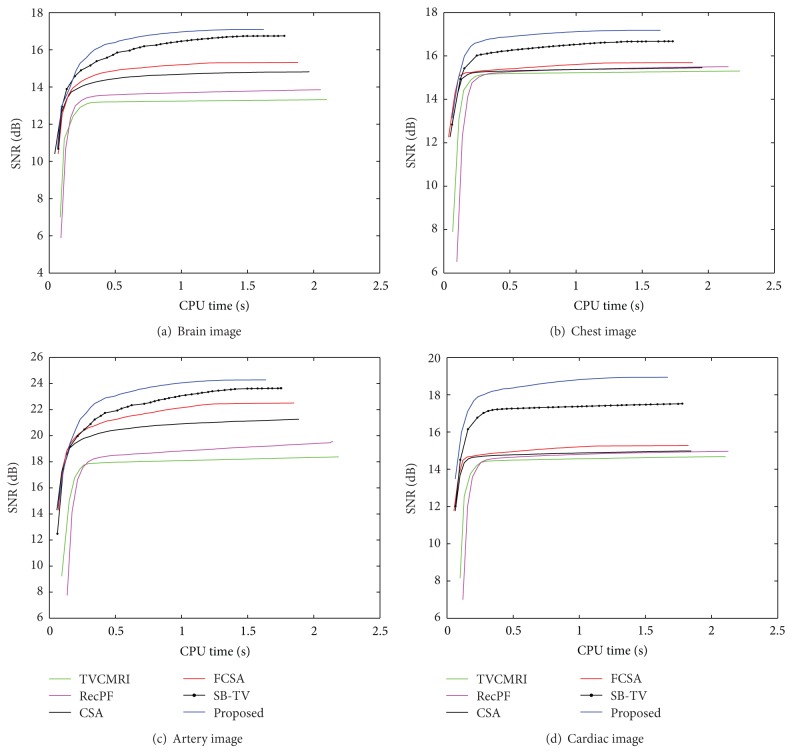
Performance comparisons (CPU time versus SNR) on different MR images (Rician noise case).

The proposed and existing algorithms are tested using four 2D MR images: brain, chest, artery, and cardiac, imges respectively, as shown in Figure 1. They have been used for validation in [7]. For convenience, they have the same size of 256 × 256. The sample ratio is set to be approximately 20%. To perform comparisons, all methods run 50 iterations, except SB-TV and the proposed method. SB-TV completes reconstruction in 35 iterations, while the proposed method takes only 30 iterations. The proposed method provides best visual effects on all MR images. Figures 2, 3, 4, and 5 show the visual comparisons of the reconstructed results using Gaussian noisy observations by different methods in the brain, chest, artery, and cardiac images, respectively.  Figure 10 gives the performance comparisons between different methods in terms of the CPU time over SNR.  Table 1 shows the average value of CPU time, SNR, RE, SSIM and FSIM of different methods in Gaussian noise case. Figures 6, 7, 8, and 9 show the visual comparisons of the reconstructed results using Rician noisy observations by different methods in the brain, chest, artery, and cardiac images, respectively.  Figure 11 gives the performance comparisons between different methods in terms of the CPU time over SNR.  Table 2 shows the average value of CPU time, SNR, RE, SSIM and FSIM of different methods in Rician noise case. In both cases, the reconstructed results produced by the proposed method is better than those produced by the TVCMRI, RecPF, CSA, FCSA, and SB-TV. The reconstruction performance of the proposed work is the best in terms of both the computational complexity and reconstruction accuracy, which clearly demonstrate the efficiency of this method for the compressed MR image reconstruction.

## 6. Conclusion

An efficient algorithm is proposed for the compressed MR image reconstruction based on nonlocal total variation and Split Bregman method. The algorithm effectively solves a NLTV-based *L*_1_-norm term by using the Split Bregman method. NLTV can effectively avoid blocky artifacts caused by traditional TV regularization. Numerous experiments were conducted to compare different reconstruction methods. The results of our study indicate that the proposed method outperforms the classic methods in terms of both accuracy and complexity.

## Figures and Tables

**Algorithm 1:**
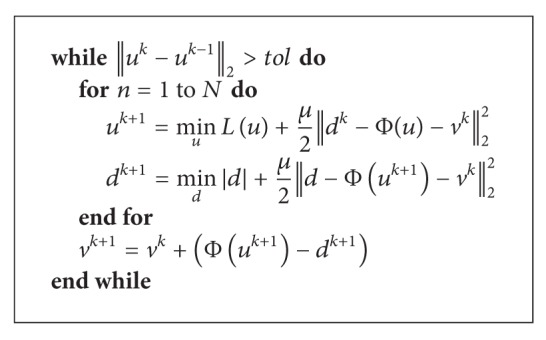
Generalized Split Bregman algorithm.

**Algorithm 2:**
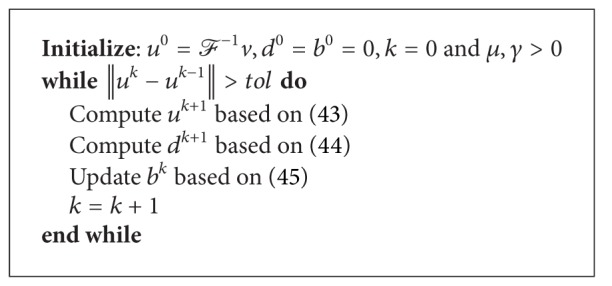
Proposed algorithm.

**Table 1:** Comparison of the average values of quality evaluation parameters (Gaussian noise case).

	Image	TVCMRI	RecPF	CSA	FCSA	SB-TV	Proposed
SNR (dB)	Brain	14.19	14.75	15.20	15.78	16.31	17.49
	Chest	16.99	17.38	17.13	17.63	18.03	18.89
	Artery	18.70	19.98	22.15	23.61	24.17	25.75
	Cardiac	16.97	17.65	17.95	18.77	18.98	19.47

RE (%)	Brain	16.96	16.54	07.97	07.37	06.90	05.98
	Chest	11.58	11.21	10.84	10.33	08.89	07.96
	Artery	12.61	11.84	05.77	04.90	04.57	04.10
	Cardiac	14.03	13.39	11.10	09.78	08.29	07.72

SSIM	Brain	0.9567	0.9742	0.9906	0.9935	0.9940	0.9947
	Chest	0.9829	0.9839	0.9848	0.9861	0.9887	0.9914
	Artery	0.9825	0.9835	0.9848	0.9919	0.9924	0.9940
	Cardiac	0.9614	0.9642	0.9748	0.9799	0.9835	0.9889

FSIM	Brain	0.8712	0.8773	0.9489	0.9654	0.9719	0.9765
	Chest	0.8990	0.9012	0.9063	0.9148	0.9237	0.9374
	Artery	0.9010	0.9168	0.9516	0.9668	0.9705	0.9742
	Cardiac	0.9313	0.9366	0.9454	0.9551	0.9587	0.9629

CPU time (s)	Brain	2.14	2.05	1.96	1.88	1.78	1.62
	Chest	2.21	2.16	1.95	1.86	1.73	1.62
	Artery	2.26	2.15	1.86	1.83	1.72	1.64
	Cardiac	2.13	2.10	1.84	1.82	1.80	1.67

**Table 2:** Comparison of the average values of quality evaluation parameters (Rician noise case).

	Image	TVCMRI	RecPF	CSA	FCSA	SB-TV	Proposed
SNR (dB)	Brain	13.31	13.85	14.81	15.31	16.24	17.08
	Chest	15.30	15.50	15.45	15.69	16.67	17.18
	Artery	18.34	19.55	21.26	22.54	23.64	24.28
	Cardiac	14.68	14.95	14.98	15.26	17.52	18.93

RE (%)	Brain	17.15	16.78	13.01	12.39	10.36	10.01
	Chest	13.19	12.94	11.58	10.98	09.38	08.81
	Artery	13.24	12.46	06.76	05.95	05.04	04.71
	Cardiac	15.06	14.73	12.69	10.24	09.89	08.96

SSIM	Brain	0.9511	0.9625	0.9787	0.9807	0.9860	0.9870
	Chest	0.9747	0.9762	0.9801	0.9816	0.9828	0.9889
	Artery	0.9810	0.9824	0.9832	0.9889	0.9904	0.9924
	Cardiac	0.9582	0.9592	0.9697	0.9717	0.9812	0.9858

FSIM	Brain	0.8499	0.8562	0.8994	0.9082	0.9290	0.9339
	Chest	0.8860	0.8968	0.9031	0.9067	0.9221	0.9298
	Artery	0.8652	0.8797	0.9424	0.9566	0.9672	0.9715
	Cardiac	0.9220	0.9304	0.9352	0.9404	0.9518	0.9565

CPU time (s)	Brain	2.10	2.05	1.96	1.88	1.78	1.62
	Chest	2.26	2.14	1.97	1.85	1.73	1.63
	Artery	2.18	2.14	1.88	1.84	1.74	1.63
	Cardiac	2.10	2.12	1.84	1.82	1.79	1.67
